# Do Primary Care Providers’ Medicaid Panels Represent the Communities They Serve?

**DOI:** 10.3390/healthcare13162062

**Published:** 2025-08-20

**Authors:** Anushree Vichare, Qian (Eric) Luo, Mandar Bodas

**Affiliations:** Fitzhugh Mullan Institute for Health Workforce Equity, Department of Health Policy and Management, Milken Institute School of Public Health, George Washington University, Washington, DC 20052, USA; avichare@gwu.edu (A.V.); qluo@gwu.edu (Q.L.)

**Keywords:** health workforce, Medicaid, underserved communities, access to care, health policy

## Abstract

Disparities in primary care access among Medicaid enrollees may be driven by differences in provider acceptance of Medicaid, yet the extent to which primary care provider (PCP) panels reflect the racial and ethnic diversity of local Medicaid populations is unknown. **Objectives**: To quantify the alignment between the racial/ethnic composition of PCP Medicaid panels and the underlying Medicaid population in their service areas. **Methods**: We conducted a cross-sectional analysis of 2019 Transformed Medicaid Statistical Information System Analytic Files from 44 states focusing on non-Hispanic White (NHW), non-Hispanic Black (NHB), and Hispanic enrollees. We calculated a panel representation ratio (PRR) for each PCP (physicians, nurse practitioners, and physician associates) as the proportion of a racial/ethnic group in their panel divided by that group’s proportion in the county Medicaid population. PRRs > 1 indicate overrepresentation; PRRs < 1, underrepresentation. Analyses were stratified by provider specialty, rurality, and Health Professional Shortage Area (HPSA) status. **Results**: The study sample included 372,320 PCPs from the following professions: nurse practitioners (NPs) and physician associates (PAs), along with physicians from the following specialties: family physicians (FPs), internal medicine physicians (IM), obstetrician gynecologists (ObGyn), and pediatricians (Peds). In the full sample, PRR was 1.28 for NHW enrollees, but less than one for NHB (0.98) and Hispanic (0.82) enrollees. Across provider specialties and professions, NHW enrollees were overrepresented in both rural and urban areas. In rural areas, NHB enrollees were overrepresented, but Hispanic enrollees remained underrepresented regardless of Health Professional Shortage Area (HPSA) status. In urban areas, both NHB and Hispanic enrollees were underrepresented in provider panels. **Conclusions**: Medicaid PCP panels do not reflect the racial/ethnic diversity of local Medicaid populations, particularly for NHB and Hispanic enrollees in urban settings. Efforts to improve equitable access to primary care must address these disparities in provider panel composition.

## 1. Introduction

Access to primary care is a cornerstone of health equity, especially for vulnerable populations such as those with disabilities or low income. In the United States, such populations are covered by a program called Medicaid [1]. Medicaid covers a wide array of groups, including low-income adults, children, pregnant women, seniors, and people with disabilities. Each state administers its own Medicaid program within federal guidelines, resulting in some variation in eligibility and benefits across the country. Research indicates that Medicaid has significantly improved recipients’ health outcomes, and financial security, and it has provided broad economic benefits to both states and health providers [2,3]. Yet Medicaid enrollees continue to face significant barriers in obtaining timely and consistent care. Numerous studies have shown that Medicaid enrollees experience greater difficulty scheduling appointments and report higher levels of unmet healthcare needs compared to those with private insurance [4,5,6]. These challenges are even more pronounced among racial and ethnic minority groups within the Medicaid population, who often encounter compounded barriers due to systemic inequities [7,8]. Addressing these disparities requires not only expanding insurance coverage but also ensuring that primary care providers (PCPs) are both available and willing to serve Medicaid enrollees [9,10].

While financial concerns such as low reimbursement rates and administrative complexity are commonly cited reasons for limited provider participation in Medicaid [11,12,13,14,15], there is growing recognition that non-financial factors may also influence provider behavior. For example, studies show that the racial and ethnic composition of a local Medicaid population may affect whether providers choose to participate in the program [16,17]. This raises important questions about how structural factors may shape access to care for Medicaid enrollees from different racial and ethnic groups. For instance, if providers in communities with high racial/ethnic diversity do not accept patients covered by Medicaid, then it is likely that the patient panels of such providers will not include individuals from diverse racial/ethnic backgrounds. In other words, the provider patient panels will not truly reflect the demographic composition of the communities they serve, potentially reinforcing disparities in access to care.

Relatively little is known about how the racial and ethnic composition of Medicaid enrollees is represented within the patient panels (referred to as Medicaid panels hereon) of PCPs [18]. Understanding this variation is essential, as it may reveal patterns of concentration or dispersion that are not immediately visible through traditional measures of access. To explore this issue, we introduce a new metric called the panel representation ratio, or PRR. This measure compares the racial and ethnic composition of a provider’s Medicaid panel to that of the surrounding Medicaid population at the county level. A PRR of one indicates proportional representation; if it is more than one, that may indicate overrepresentation, and if it is below one, that indicates underrepresentation.

While this study focuses on Medicaid in the United States, similar challenges in ensuring equitable access to care exist in other countries with publicly funded or mixed healthcare systems [19]. Understanding how provider participation intersects with the racial and ethnic composition of publicly insured populations is therefore relevant to broader international efforts to ensure equitable access to care [20]. The concept of PRR can provide a useful lens to examine similar dynamics in other healthcare systems.

Using multi-state Medicaid claims data, we estimated PRRs for a large sample of PCPs. We examined how PRRs vary by provider specialty, geographic location, Health Professional Shortage Area (HPSA) designation, and practice setting. Special attention was given to Community Health Centers (CHCs), which play a critical role in serving Medicaid populations. CHCs are critical providers for Medicaid populations, offering comprehensive, culturally responsive, and accessible primary care services. They serve millions of Medicaid enrollees each year, including racial and ethnic minorities, people with disabilities, and those in medically underserved areas [21]. Our goal was to identify patterns that can inform more equitable workforce policies and improve access to care for all Medicaid enrollees.

## 2. Materials and Methods

We used multi-state Medicaid claims data from the 2019 Transformed Medicaid Statistical Information System (T-MSIS) Analytic Files (TAF) in a cross-sectional design to determine PCPs’ Medicaid panels. PCPs included nurse practitioners (NPs), physician associates (PAs), and physicians practicing in family physician (FP), general internal medicine (IM), obstetrics and gynecology (ObGyn), and pediatrics specialties. The National Plan and Provider Enumeration System (NPPES) was used as the primary source for clinician location and specialty classification [22].

We obtained the race and ethnicity of enrollees in PCPs’ Medicaid panels and categorized them as non-Hispanic White (NHW), non-Hispanic Black (NHB), non-Hispanic Asian (NH Asian), non-Hispanic American Indian/Alaska Native (NH AIAN), non-Hispanic Hawaiian/Pacific Islander (NH HPI), and Hispanic. Next, we used the demographic and eligibility (DE) file from TAF to determine the racial and ethnic composition of the Medicaid population in each county. A patient was included in a particular PCP’s panel if the PCP provided any outpatient service to that patient. For each PCP, we then created a panel representation ratio (PRR) for each racial and ethnic group. We calculated this by dividing the percentage of a specific group in the provider’s Medicaid panel by that group’s percentage in the county’s overall Medicaid enrollee population. A PRR of one indicates that the group is proportionally represented in the provider’s panel, with values more or less than one suggesting overrepresentation or underrepresentation, respectively. We recognize that the county of a PCP’s practice location may not always serve as the most accurate catchment area for defining the community served by that PCP, as patients may cross county lines or be drawn from broader geographic regions [23]. However, developing refined catchment area measures was beyond the scope of this study. Therefore, we used the Medicaid population of a PCP’s county as a proxy for the community they served.

The calculation of PRR for NHW enrollees can be represented as

(1) $$PRR^{NHW}=\frac{\%\;NHW\;in\;a\;PCPs\;Medicaid\;Patient\;Panel}{\%\;NHW\;in\;county\;Medicaid\;population}$$

Equation (1) is an example of *PRR^NHW^* calculation for PCP Medicaid panels.

While data were available for six racial and ethnic groups, our primary analyses focused on NHW, NHB, and Hispanic enrollees. These groups represented the largest segments of the Medicaid population and allowed for more stable estimates across geographic areas. To avoid skewing our results, we excluded PCPs with extremely large (more than 5000 patients) or very small (fewer than 10 patients) Medicaid enrollee panels. We also excluded PCPs whose panels had more than 50% of enrollees with missing race or ethnicity data. Additionally, providers practicing in states with poor quality T-MSIS Analytic Files (TAF) data were excluded. As described in prior workforce studies using TAF, data quality was assessed by examining the proportion of claims from each state that lacked provider identifiers [24]. Based on this criterion, six states (Delaware, Florida, Maine, Minnesota, New Hampshire, and Rhode Island) were excluded. The final analytic sample included data from the remaining 44 states.

We examined panel diversity and PRR across four key dimensions: (1) provider profession/specialty, (2) rurality of the county (classified using the USDA’s Rural–Urban Continuum Codes (RUCCs)), (3) geographic Health Professional Shortage Area (HPSA) designation, and (4) practice setting, specifically whether the provider worked at a CHC. PCPs in rural areas may exhibit distinct practice patterns not typical in densely populated and more diverse urban areas. HPSA designation is used to identify areas with shortages of PCPs and to allocate resources such as loan repayment programs and workforce incentives. Including HPSA status in the analysis allows us to assess whether these designations align with equitable representation of racial and ethnic groups in provider panels. Finally, CHCs are the main safety net providers for Medicaid populations and are likely to have unique practice patterns. We identified all providers associated with CHCs using methods described elsewhere [25].

To assess whether provider Medicaid panels in each subgroup (e.g., HPSA vs. non-HPSA, rural vs. urban, or by specialty) differed meaningfully from the overall sample, we conducted a series of *t*-tests. For each variable of interest, we compared the mean within each subgroup to the overall sample mean. This approach allowed us to identify which providers had Medicaid panels that were significantly more or less representative of specific racial and ethnic populations compared to the full sample. All analyses were conducted using Stata 18.

## 3. Results

### 3.1. Sample Description

Among the 372,320 PCPs in the sample, 22.1% practiced in Health Professional Shortage Areas (HPSAs), and 7.0% practiced in rural HPSAs (Table 1). Rural providers overall accounted for 7.1% of the total sample. Compared to those not in HPSAs, providers in HPSAs served panels with a higher proportion of NHW enrollees (67.7% vs. 50.7%) and a lower proportion of NHB (17.6% vs. 21.5%) and Hispanic enrollees (10.1% vs. 20.2%). Rural HPSA panels were the least diverse, with 78.2% NHW enrollees and only 8.2% NHB enrollees. PRRs showed consistent overrepresentation of NHW enrollees (range: 1.04 to 1.32) and underrepresentation of Hispanic enrollees (range: 0.80 to 1.03) across all groups. Comparisons of panel sizes revealed interesting patterns—the average panel size was the largest among rural HPSA providers (339) and the smallest among urban non-HPSA providers (291).

### 3.2. Specialty Comparison

Across provider types, pediatricians had the largest and the most diverse panels, with the lowest proportions of NHW enrollees (43.4%) and the highest proportions of Hispanic enrollees (26.8%) (Table 2). ObGyns followed a similar pattern, with 46.4% NHW and 21.2% Hispanic enrollees in their panels. In contrast, other providers had higher shares of NHW enrollees (around 58%). PRRs showed consistent overrepresentation of NHW enrollees across all provider types (range: 1.12 to 1.39), while Hispanic enrollees were generally underrepresented, except in pediatrics (PRR: 1.17).

### 3.3. Comparison of CHC and Other PCPs

**Figure 1:**
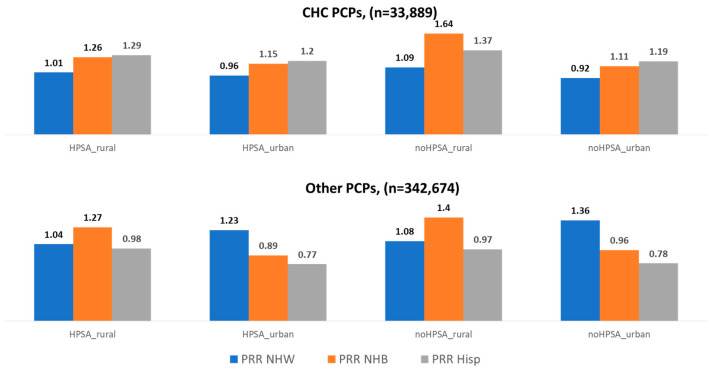
Comparing the PRR of PCPs associated with CHCs (**top** panel) to the PRRs of other PCPs (**bottom** panel), TAF 2019. HPSA_rural = PCP practiced in a rural county that was an HPSA; HPSA_urban = PCP practiced in an urban county that was an HPSA; noHPSA_rural = PCP practiced in an rural county that was not an HPSA; noHPSA_urban = PCP practiced in an urban county that was not an HPSA; PRR NHW = panel representation ratio, Non-Hispanic White; PRR NHB = panel representation ratio, Non-Hispanic Black; PRR Hisp = panel representation ratio, Hispanic.

Patterns of PRR varied notably between those practicing at CHCs and other settings (Figure 1). CHC providers consistently served more racially and ethnically representative panels, with PRRs for NHB and Hispanic enrollees exceeding 1.0 across all geographic and HPSA categories, reaching as high as 1.64 for NHB and 1.37 for Hispanic enrollees in non-HPSA rural areas. In contrast, other PCPs showed underrepresentation of Hispanic enrollees in all settings, with PRRs ranging from 0.77 to 0.98, and mixed representation for NHB enrollees, with overrepresentation in rural areas but underrepresentation in urban HPSAs. NHW enrollees were generally overrepresented among other PCPs, especially in urban non-HPSAs where the PRR reached 1.36, while CHC providers showed more balanced NHW representation with PRRs near or less than 1.0. These findings highlight CHCs’ stronger alignment with the racial and ethnic composition of their communities, particularly in underserved areas.

## 4. Discussion

To our knowledge, this is the first study that estimates the racial and ethnic diversity of PCPs’ Medicaid enrollee panels using multi-state claims data. We find that ObGyns and pediatricians had higher proportions of Hispanic enrollees in their panels. This is consistent with both a relatively higher proportion of Hispanic births funded by Medicaid compared to other subgroups, and a higher proportion of Hispanic children among those covered by Medicaid.

Using the PRR, our findings show that enrollee panels are racially and ethnically more diverse in urban non-HPSA. These findings remain consistent regardless of PCP specialty. However, despite the higher proportion of racial and ethnic minorities in enrollee panels of urban PCPs, this did not translate into higher representation relative to their share of the county’s Medicaid population. One possible explanation is that NHB and Hispanic Medicaid enrollees may be less likely to see PCPs than other types of providers, such as those in emergency room settings. The literature suggests a preference for non-primary care sites as a usual source of care, particularly among NHB individuals from low-income, urban, and racially integrated areas, which has been attributed to medical mistrust [26]. Additionally, underrepresentation in enrollee panels may reflect barriers to care faced by enrollees from racial ethnic minority groups. Racial and ethnic disparities in determinants of access, such as lack of transportation, long waiting times, and inconvenient office hours, have worsened over time [27]. These factors, combined with difficulties in obtaining appointments due to low Medicaid acceptance, can further impede access [11,28,29,30]. Regardless of the cause, limited access to or use of primary care by marginalized communities is concerning, as it can lead to poorer health outcomes, preventable hospitalizations, and higher costs of care [26].

It is also noteworthy that we find a higher representation of NHB and Hispanic Medicaid enrollees in the panels of PCPs practicing at CHCs, regardless of the CHCs’ rurality or HPSA designation. One plausible explanation is that Medicaid enrollees may prefer seeking care at CHCs. Alternatively, this pattern could reflect greater conscious or unconscious bias among non-CHC PCPs, or a shortage of bilingual providers in those settings [31]. Regardless of the reason, the findings suggest that non-CHC PCPs and settings could improve their inclusivity, particularly with respect to Hispanic enrollees. These findings add to a growing body of evidence on the pivotal role of CHCs in providing primary care to racial and ethnic communities [32,33], and supports arguments that greater investment in CHCs could improve access to care for low-income and minority individuals [34].

Our study has several limitations. First, TAF data are reported at the state level, and some states have insufficient data on enrollee race and ethnicity and provider characteristics. Second, we relied on a single year of data to calculate panel diversity, though we recognize that panel composition may change over time. Third, we were only able to identify PCPs associated with CHCs when services were billed under an individual NPI. We did not control for the health conditions in the populations served by PCPs. Our data was from 2019 (before the COVID-19 pandemic) and may not fully reflect the current healthcare environment. Additionally, we acknowledge that Medicaid panels are shaped not only by individual providers but also by the practices or organizations they work for. We did not control for practice-level factors beyond PCPs’ association with CHCs. Our definition of a PCP’s catchment area was quite broad and did not account for factors such as local-level transportation. Future analysis on panel representativeness should include alternate definitions of catchment areas such as patients living within certain distance from PCP locations or within certain travel times. Finally, this is a descriptive analysis of a novel method to assess the representation of Medicaid enrollee groups in provider panels. Future studies should examine whether this method of examining representation impacts access and health outcomes.

Our results have implications for policymaking on medical education and efforts focused on the distribution of the health workforce. Medical education curriculum can benefit from discussions on implicit biases and its impact on PCPs’ panels and on the communities they serve [35]. To improve equitable distribution of providers and the PRR of enrollee panels, workforce allocation policies may need to move beyond current shortage designations and incorporate additional equity-focused metrics. For example, incorporating PRR data into shortage designations could help target resources more effectively. Next, efforts can be made to increase transparency and visibility about the representativeness of PCP panels. This may create a positive feedback loop where PCPs can review their PRRs and will then attempt to improve them.

Finally, recent policy provisions which reduce Medicaid funding can potentially reduce coverage and disproportionately affect low-income and minority populations, straining access to primary care [36]. As policymakers debate the future of Medicaid, tools like the PRR can help ensure that equity considerations inform workforce and access planning. The recently published final rule on Medicaid managed care establishes new standards for access, transparency, and quality, including benchmarks for wait times and enrollee experience surveys [37]. Evaluations of these policies and regulations could include active monitoring of the racial and ethnic representation in PCPs’ Medicaid panels using the PRR methodology.

## 5. Conclusions

Inequitable workforce distribution remains a persistent health policy challenge. Deep-rooted discriminatory policies in the United States have exacerbated these distributional inequities, particularly in racially segregated areas [38]. Policies aimed at universal access have traditionally focused on redistributing the workforce by incentivizing PCPs to practice in designated shortage areas [39]. However, our findings suggest that marginalized communities in non-shortage urban areas may face even greater barriers than those in rural or shortage areas, and that CHCs play a critical role in serving diverse populations.

## Figures and Tables

**Table 1:** Characteristics of Medicaid panels of primary care providers in the sample.

	Full Sample	Not Practicing in HPSAs	Practicing in HPSAs	HPSA Rural	HPSA Urban	Non-HPSA Rural	Non-HPSA Urban
PCPs (N)	372,320	289,884	82,436	13,035	69,401	12,998	276,886
Panel characteristics *							
% NH White enrollees ^$^	54.5	50.7 **	67.7 **	78.2 **	65.8 **	69.7 **	49.8 **
% NH Black enrollees	20.6	21.5 **	17.6 **	8.2 **	19.4 **	11.6 **	21.9 **
% NH Asian enrollees	4.2	4.9 **	1.6 **	0.4 **	1.9 **	0.5 **	5.2 **
% NH AIAN enrollees	1.8	1.7 **	2.1 **	4.3 **	1.6 **	8.0 **	1.4 **
% NH HPI enrollees	0.6	0.7 **	0.2 **	0.1 **	0.3 **	0.2 **	0.7 **
% Hispanic enrollees	17.9	20.2 **	10.1 **	7.9 **	10.5 **	9.1 **	0.4 **
% Enrollees missing race/ethnicity data	11.9	11.2 **	14.4 **	9.2 **	15.3 **	9.0 **	20.7 **
Panel Representative Ratios (PRRs)							
PRR: NH White	1.28	1.31 **	1.18 **	1.04 **	1.21 **	1.08 **	1.32 **
PRR: NH Black	0.98	0.99	0.97	1.28 **	0.91 **	1.44 **	0.97 **
PRR: Hispanic	0.82	0.82	0.84	1.02 **	0.80 **	1.03 **	0.81 **
Average panel size	298	292	318	339	314	315	291

* *p* < 0.05, ** *p* < 0.01, ^$^ NH = non-Hispanic; AIAN = American Indian/Alaska Native; HPI = Hawaiian/Pacific Islander.

**Table 2:** Comparison of Medicaid panels by PCPs’ specialty and profession.

	NPs	PAs	FM	IM	ObGyn	Pediatricians
PCPs (N)	125,221	56,647	74,864	51,251	27,088	37,249
Panel characteristics *						
% NH White enrollees ^$^	57.5 **	58.0 **	58.0 **	50.2 **	46.4 **	43.4 **
% NH Black enrollees	20.5	17.6 **	17.3 **	25.3 **	25.5 **	22.2 **
% NH Asian enrollees	3.0 **	3.5 **	4.3 *	7.2 **	4.4 **	4.9 **
% NH AIAN enrollees	1.9 **	2.1 **	2.1 **	1.2 **	1.5 **	1.3 **
% NH HPI enrollees	0.5 **	0.4 **	0.6 **	0.6	0.6 **	0.8 **
% Hispanic enrollees	16.1 **	17.8	17.3 **	15.4 **	21.1 **	26.8 **
% Enrollees missing race/ethnicity data	11.4 **	11.1 **	10.9 **	12.2 **	11.0 **	16.6 **
Panel Representative Ratios (PRRs)						
PRR: NH White	1.28	1.32 **	1.31 **	1.39 **	1.18 **	1.12 **
PRR: NH Black	0.96 **	0.94 **	0.98	1.04 **	1.12 **	0.97
PRR: Hispanic	0.81 **	0.80 **	0.77 **	0.66 **	0.92 **	1.17 **
Average panel size	256	300	205	259	292	626

* *p* < 0.05, ** *p* < 0.01, ^$^ NH = non-Hispanic; AIAN = American Indian/Alaska Native; HPI = Hawaiian/Pacific Islander.

## Data Availability

The data used in this study was obtained by authors from the Centers for Medicare and Medicaid Services (CMS) the data use agreement signed by the authors disallows them from sharing it.

## Abbreviations

The following abbreviations are used in this manuscript:

PCP	Primary Care Provider
CHC	Community Health Center
CMS	Centers for Medicare and Medicaid Services
NHW	Non-Hispanic White
NHB	Non-Hispanic Black
TAF	Transformed Medicaid Statistical Information System Analytic Files (TAF)
NPPES	National Plan and Provider Enumeration System
NPI	National Provider Identifier
NP	Nurse Practitioner
FP	Family Physician
IM	Internal Medicine
ObGyn	Obstetrics/Gynecology
PA	Physician Associates
